# Holes can be perilous: A rare presentation of intestinal obstruction - Herniation through the broad ligament

**DOI:** 10.1016/j.radcr.2023.12.067

**Published:** 2024-01-13

**Authors:** Yung Kuan Moses Wong, Wei Woon Teng, Zi Ching Sharon Chong, Chia Shing Tan, Yue Yuan Wong, Danesh Thangavelu, Muhammad Danial Md Umar, Firdaus Hayati

**Affiliations:** a Department of Surgery, Queen Elizabeth Hospital, Ministry of Health Malaysia, Kota Kinabalu, Sabah, Malaysia; b Department of Radiology, Keningau Hospital, Ministry of Health Malaysia, Keningau, Sabah, Malaysia; c Department of Surgery, Faculty of Medicine and Health Sciences, Universiti Malaysia Sabah, Kota Kinabalu, Sabah, Malaysia

**Keywords:** Broad ligament hernia, Internal hernia, Intestinal obstruction

## Abstract

A 57-year-old lady presented with symptoms of intestinal obstruction preceded by a 2-day history of lower abdomen pain. Clinically, she had a distended abdomen with tenderness in her lower abdomen. Laboratory investigations were unremarkable. An abdominal radiograph showed a dilated small bowel with no extensive bowel gas. A computed tomography (CT) scan showed suspected intestinal obstruction secondary to herniation into the right broad ligament. The decision was made to proceed with surgery, and the intraoperative results confirmed the CT results. The literature review is outlined here, and this instance illustrates a surprising discovery.

## Introduction

Intestinal obstruction is commonly encountered in our practice. However, internal hernia figures among its rare causes and accounts for only around 1%-2% of all cases [1]. An internal hernia is described as an intra-abdominal viscera protruding via a peritoneal or mesentery defect. The cause of such defects can be either congenital or iatrogenic. Only 4% of all internal hernias are caused by herniation via the wide ligament, which is seldom documented [2]. Patients with broad ligament herniation might present with a history resembling acute appendicitis or, in the more serious form, intestinal obstruction symptoms, namely vomiting, abdominal distension, and an inability to pass flatus. Since there are no characteristic symptoms or signs, preoperative diagnosis is therefore challenging [3]. During surgical investigation, the majority of patients with a wide ligament have been identified [4]. Herein, we present a case of small bowel obstruction with strongly suspected internal herniation through the board ligament of the uterus, which was diagnosed preoperatively with CT and managed laparoscopically.

## Case presentation

A 57-year-old lady presented with lower abdominal pain mostly at the suprapubic and right iliac fossa for 3 days. She sought medical attention and was treated for a urinary tract infection with oral antibiotics. She then complained of worsening abdomen distension, no bowel opening, and multiple vomiting 1 day before admission. She had no suspicious bowel symptoms before this. She denied a history of tuberculosis and claimed no family history of colon malignancy. However, she had undergone a laparoscopic cholecystectomy 5 years previously due to a gallstone-related complication.

**Fig. 1:**
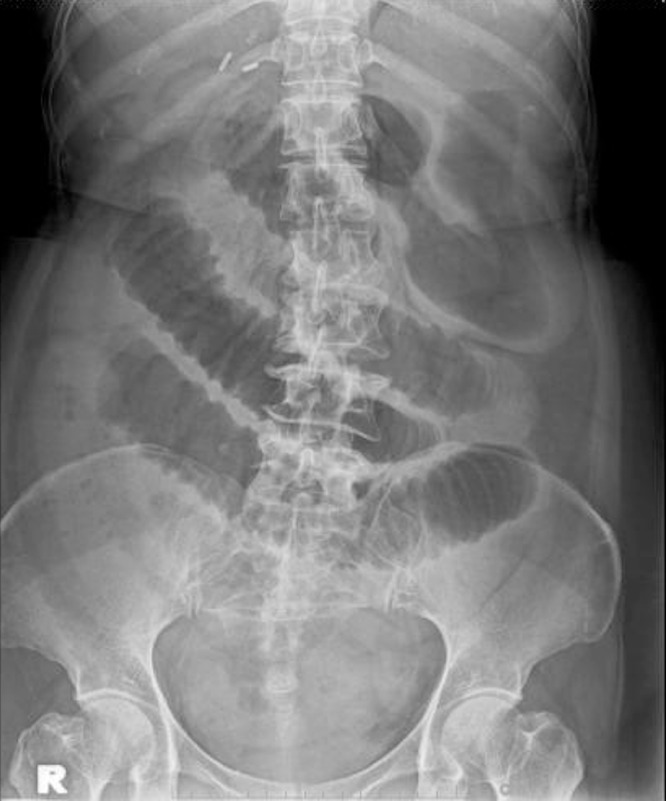
Abdominal radiograph showed a dilated small bowel with no air in the large bowel.

**Fig. 2:**
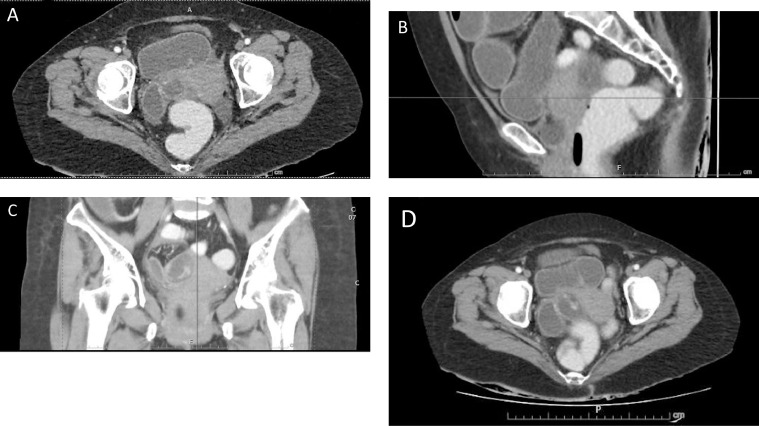
CT of the abdomen at axial (A), coronal (B) and sagittal (C) view showed a short segment of narrowing at the distal ileum (17 cm from ileocecal junction) positioned immediately inferior to the right fallopian tube.

On examination, the abdomen was distended. Her suprapubic and right iliac fossa were tender with voluntary guarding. Her laboratory investigations were normal. Abdominal radiographs showed a dilated small bowel with no extensive bowel gas (Fig. 1). We proceeded with a computed tomography (CT) of the abdomen. Unexpectedly, this revealed a possible small bowel internal herniation at the right wide ligament (Fig. 2). It showed a short segment of narrowing at the distal ileum (17 cm from the ileocaecal junction) positioned immediately inferior to the right fallopian tube.

**Fig. 3:**
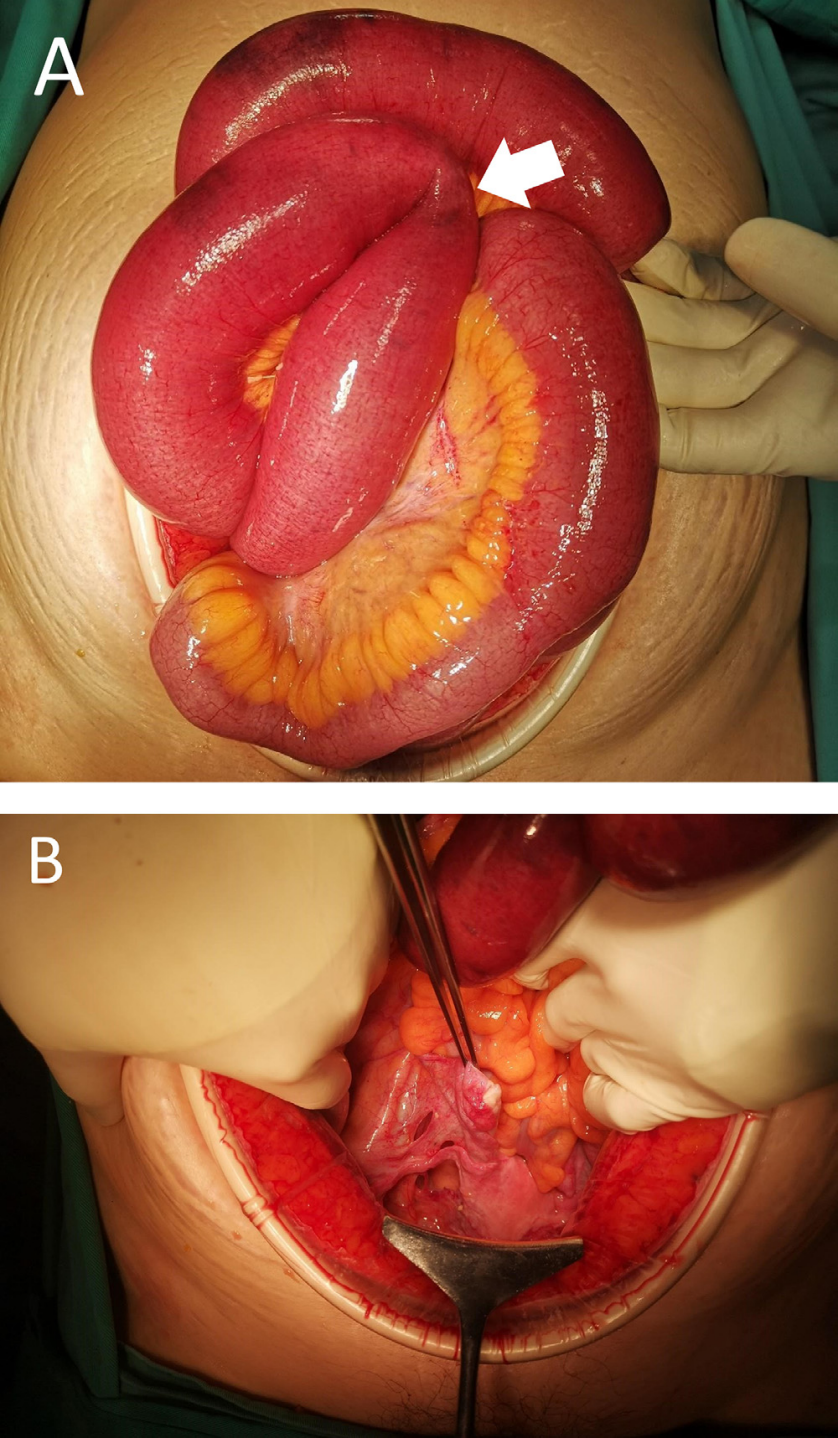
(A) A dilated small bowel with transitional point noted (white arrow). (B) A total of 3 defects were seen on the right broad ligament.

**Fig. 4:**
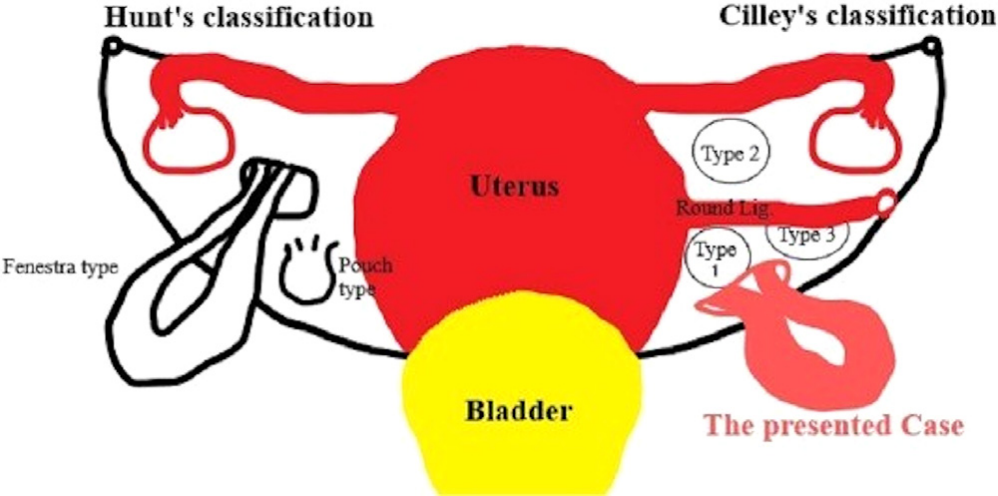
Cilley's broad ligament herniation types.

We proceeded with an open exploration of the abdomen under general anesthesia. Upon entry, intraoperatively, we noticed minimal ascites with small bowel dilatation from the duodenojejunal junction up to the distal ileum (Fig. 3). We discovered that a short segment of the distal ileum was herniated through one of the 3 defects on the right broad ligament (Fig. 4). With particular care, the herniated small bowel could be released with ease. We then decompressed 1.8 L of the small bowel content through the nasogastric tube and primarily repaired the broad ligament defects with absorbable sutures. The left broad ligament was normal, as were the other visceral organs. She made a slow recovery and was discharged uneventfully.

## Discussion

Ghahremani and Meyers [5] categorized internal hernias based on where the hernia orifice is located and stated they are extremely uncommon, with a reported incidence of less than 1% . Within the internal hernias category, paraduodenal hernias account for 50% of cases, pericecal hernias account for 13%, and hernias via the foramen of Winslow account for 8% [6]. Despite accounting for about 4%-7% of all internal hernias, herniation via the wide ligament is extremely uncommon.

Quain first documented broad ligament herniation in an autopsy series in 1861 [7]. It can be categorized using any of the 2 Cilley or Hunt categories. In 1986, Cilley classified wide ligament abnormalities into 3 categories: type 1 flaws occur caudal to the round ligament; type 2 defects occur above the round ligament of the uterus; and type 3 defects occur in the mesoligamentum teres of the uterus (Fig. 4) [8]. Hunt classified it into 2 types, fenestra and pouch, based on the nature of the defect (Fig. 4) [9].

Both congenital and acquired defects can affect the wide ligament. Surgery, pelvic inflammatory illness, and pregnancy-related trauma are examples of acquired causes. Cystic formations inside the wide ligament, believed to represent congenital remains of the mesonephric or Mullerian ducts, may spontaneously rupture in nulliparous individuals, resulting in such abnormalities [8]. When evaluating a patient who has acute symptoms of intestinal blockage and no external hernias, congenital internal hernias should be taken into account, despite their rarity [10]. Apart from a thorough history and clinical examination, radiographs such as abdominal radiography or ultrasonography (USG) might lead practitioners to diagnose intestinal obstruction. However, a contrasted CT scan has the advantage of delineating the pathology. Making an early diagnosis is deemed crucial to prevent complications such as ischemia, strangulation, and perforation.

In this case, a CT scan showed dilated bowels with a transition point over the distal jejunum with strongly suspected herniation through the broad ligament of the uterus. It might be difficult to determine the cause of the transition point at times, even with CT scans, because herniation through the broad ligament is clinically rare. Nevertheless, we were able to obtain a preoperative diagnosis in which the preoperative planning and approach were well designed. Intraoperative management is comprised of 2 parts. First, using the Trendelenburg position, the incarcerated contents are gently reduced, and, if necessary, the nonviable bowel is resected. Second, to prevent recurrent small-bowel obstruction, the defect can be closed or the broad ligament can be completely divided [4,11]. Compared to open surgery, laparoscopic surgery is recognized to offer several benefits, including a lower infection rate, better cosmetic results, less discomfort, and a shorter recovery period [12].

## Conclusion

When a female patient presents with abdominal pain with or without symptoms of intestinal obstruction, a high clinical suspicion for broad ligament herniation should be instilled, while more common causes like neoplasm, adhesions, and inguinal hernia should be excluded. Given its rare occurrence and nonspecific clinical presentation, a contrasted CT scan would help make life-saving decisions. Adequate resuscitation and early surgical intervention reduce the morbidity of patients. Intraoperatively, it is important to inspect the broad ligament fully, reduce the herniated content, and fix the defects to avoid future complications. The laparoscopic approach should be considered as it has advantages compared to open surgery in terms of cosmesis, shorter recovery times, and shorter hospital stays.

## Patient consent

Written informed consent for the publication of this case report was obtained from the patient.
